# Fire in Hospitals

**Published:** 1909-12-25

**Authors:** J. Compton Merryweather

**Affiliations:** 4 Whitehall Court, London, S. W.


### Editor's Letter-Box.

### FIRE IN HOSPITALS.

*To the Editor of* The Hospital.

Sir,—I have been interested to read the correspondence on the above subject appearing in your issue of the 11th inst., but do not agree with Mr. Howard J. Collins that fire drill should be confined to the male servants. At such premises as hospitals there are, of course, nurses and numerous female assistants, and I certainly consider that all such persons should have periodical drill with the fire appliances. Such a course is adopted at a large number of hospitals and other public institutions, and is undoubtedly advisable, seeing that in such buildings the female element usually predominates, so that the chances are largely in favour of an outbreak of fire being first discovered by one of the female staff. I know of several instances in which prompt action by nurses who have been trained in the use of the fire appliances, has averted what might have been a serious disaster. At stated intervals this periodical fire drill should be carried out under the superintendence of an outside expert. This ensures keeping the staff and the appliances always in proper fighting trim, so that they are likely to be more dependable in case of emergency.

Yours faithfully,

J. Compton Merryweather.

4 Whitehall Court, London, S.W., December 15, 1909.

THE HOSPITAL December 25, 1909.

